# Minimally Invasive Percutaneous Nephrolithotomy versus Retrograde Intrarenal Surgery for Upper Urinary Stones: A Systematic Review and Meta-Analysis

**DOI:** 10.1155/2017/2035851

**Published:** 2017-05-03

**Authors:** Hongyang Jiang, Zhe Yu, Liping Chen, Tao Wang, Zhuo Liu, Jihong Liu, Shaogang Wang, Zhangqun Ye

**Affiliations:** Department of Urology, Tongji Hospital, Tongji Medical College, Huazhong University of Science and Technology, Hubei 430030, China

## Abstract

Minimally invasive percutaneous nephrolithotomy (mini-PCNL) and retrograde intrarenal surgery (RIRS) are both alternatives for PCNL to treat renal calculi. This study is aimed at comparing the stone-free rate (SFR) and other surgery parameters of two approaches for treating upper urinary calculi. We performed this meta-analysis in September 2016 by searching studies about mini-PCNL and RIRS for treating upper urinary calculi in various databases, and RevMan v.5.3 was applied. Three randomized controlled trials and ten nonrandomized trials were included, involving a total of 1317 patients. Meta-analysis showed that mini-PCNL group led to a higher SFR [odds ratio: 1.96; 95% confidence interval: 1.46–2.64; *P* < 0.00001] but brought a larger postoperative decrease in hemoglobin levels compared with RIRS. RIRS provided a shorter hospital time. There was no significant difference in operation time. Higher postoperative complications were detected in the mini-PCNL, but the difference was not significant. Grade I and III complications did not vary between two procedures, but grade II complications were of lower incidence in RIRS group. In the light of these results, compared with RIRS, mini-PCNL provided significantly higher SFR and efficiency quotient for managing calculi; however, it resulted in higher incidence of postoperative complications, larger hemoglobin drops, and longer hospital stay.

## 1. Introduction

Kidney calculi is a common urological disorder which is characterized by high recurrence rate [1]. The stone movement leading to renal colic and the obstruction by calculi could result in kidney function loss. Recently, the incidence of kidney calculi has been on the rise in China, probably caused by the changed climate and environment. For releasing the obstruction, urologists choose different treatments for different size calculi diameter from less than 0.6 cm to more than 3.0 cm. As the guidelines recommend, percutaneous nephrolithotomy (PCNL), of which standard access tracts are 24–30 French (Fr), is a recommended management of patients with renal or ureteral stones more than 20 mm or and for smaller stones (10–20 mm) of the lower pole stones when anatomic factors make extracorporeal shockwave lithotripsy (ESWL) unfavorable. Although PCNL is suggested as a standard method for its excellent stone-free rate, there is still few surgical drawbacks that may compromise its efficacy [1].

For reducing postoperative morbidity associated with large devices such as blood loss, fever, and potential renal damage, minimally invasive tract has been applied widely. Minimally invasive PCNL (also termed mini-PCNL or mini-Perc or mPCNL), a miniature endoscope via a small percutaneous tract (11–20 Fr), is widely executed in the recent years [2, 3]. Mini-PCNL was described by Helal et al. Firstly performed on a 2-year-old child by the use of instruments with smaller access diameters in 1997 and developed by Jackman et al. to be a therapy option for adults [4, 5] compared to the standard tract PCNL, mini-PCNL has a more gracile tract of <20 Fr, which leads to less nephron loss and other postoperative complications; meanwhile the stone-free rate seems to have no significant difference [6, 7].

On the other hand, retrograde intrarenal surgery (RIRS) (also termed flexible ureterorenoscopy, F-URS), is another major minimally invasive measure for managing the upper urinary calculi. For its characteristics of little trauma, quick recovery, easy operation, and little contraindication, RIRS has been considered as an alternative for the percutaneous approaches for lower pole stones treatment [8, 9]. RIRS is a safe procedure with lower complication rates, blood loss, shorter length of stay, and lower stone-free rate than PCNL [10].

Mini-PCNL and RIRS are two effective minimally invasive approaches to release the obstruction. For the question of which one should be the better choice to replace the standard tract PCNL, there is not yet enough high-quality data to provide evidence. Therefore, we conducted this systematic review and meta-analysis of available literatures comparing SFR and other surgery-related parameters of mini-PCNL to RIRS for the treatment of kidney calculi.

## 2. Materials and Methods

### 2.1. Studies Selection

This meta-analysis was performed in September 2016 using PubMed, Cochrane Library, Embase, and Web of Science databases to identify related studies in accordance with the meta-analysis (PRISMA) guidelines (http://www.prismastatement.org) and preferred reporting items for a systematic review. Search strategy was as follows: (kidney stone OR urolithiasis OR kidney calculus OR kidney calculi OR renal stone OR nephrolith OR renal calculus) AND (mini-PCNL OR mPCNL OR minimally invasive surgery OR minimally invasive percutaneous nephrolithotomy OR minipercutaneous OR miniaturized PCNL OR ultra-mini-PCNL) AND (retrograde intrarenal surgery OR RIRS OR flexible ureteroscopy OR flexible ureterorenoscopy OR retrograde ureterolithotripsy).

Before the study search, we circumscribed inclusion criteria including (1) patients with kidney calculi, (2) the age >18, (3) comparing mini-PCNL with RIRS, (4) reporting at least one of the following outcomes (operative time, SFR, hemoglobin drop, hospitalization time, or postoperative complications), and (5) related parameters that could be obtained from the studies. And exclusion criteria were as follows: (1) nephrostomy tract size in mini-PCNL >20 F or <11 F; (2) conference abstracts which were not deemed to be methodologically appropriate; (3) non-English papers; (4) the inclusion criteria that were not met. Two authors accomplished the review process independently. A third author arbitrated disagreements in data extraction by consensus.

### 2.2. Data Quality Assessment

As shown in Table 1, we rated the level of evidence (LE) of every included trail according to the Oxford Centre for Evidence-Based Medicine Criteria [24]. The qualities of nonrandomized controlled trials (non-RCTs) were assessed according to Newcastle-Ottawa Scale (NOS), and RCTs qualities were assessed by the Jadad scale [25, 26]. Two reviewers performed the procedure independently and all disagreements were resolved by consensus.

### 2.3. Data Extraction and Statistical Analysis

All meta-analyses were performed to assess the overall outcomes using Review Manager Software (RevMan v.5.3, Cochrane Collaboration, Oxford, UK). Extracted data for the analysis included stone-free rate, operative time, hemoglobin drop, length of hospital stay, and postoperative complication rate. If standard deviations were not reported we estimated data according to Hozo SP [27], and if standard deviation (SD) could not be calculated from available data, we asked authors to supply the data. For evaluating dichotomous variables, we chose odds ratio (OR), and, for continuous parameters, weighted mean difference (WMD) or standardized mean difference was used. The Cochrane chi-square test and inconsistency (*I*^2^) were used to evaluate the heterogeneity among studies. Pooled estimates were calculated with the fixed-effect model for stone-free rate, and the other outcomes were calculated with the random-effect model due to the heterogeneity. Funnel plots were generated using RevMan v.5.3 to evaluate published bias of literatures. In addition, we included high-quality studies (RCTs and high score non-RCTs (NOS: 7 of 9 points)) into meta-analysis for sensitive analysis.

## 3. Results

### 3.1. Study Characteristics and Quality

**Figure 1:**
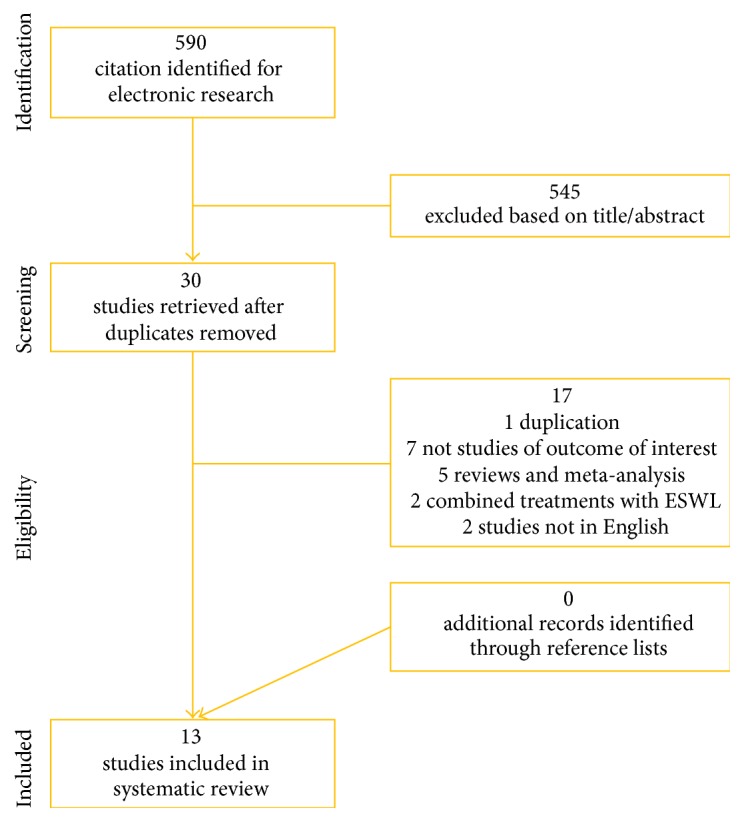
Study flow chart.

Thirteen trails were selected for analysis including a total of 701 mini-PCNL cases (53.2%) and 616 RIRS cases (46.8%) in our meta-analysis. The literature screening process is shown in Figure 1.

The baseline characteristics of the included studies are shown in Tables 2 and 3. Surgical technique for mini-PCNL differed in aspect of tract size and lithotripsy. As shown in Table 1, the studies consisted of three RCTs (LE: 2b) [11, 16, 22], two matched-pair analysis trails (LE: 3b) [13, 20], and eight case control studies (LE: 3b) including 4 prospective [14, 17, 21, 23] and 4 retrospective studies [12, 15, 18, 19]. In terms of the methodological quality, eight and two of the nonrandomized studies were relatively high (NOS: 7 of 9 points and 6 of 9 points) and medium (NOS: 5 of 9 points and 4 of 9 points), respectively. The three RCTs were of medium quality (Jadad scale: 3 of 5 points). In 3 studies, mini-PCNL and RIRS were compared between patients with a single stone. There were also two studies comparing mini-PCNL with RIRS for proximal ureteral stones, whereas the rest were compared for renal calculi. In general, the preoperative demographic characteristics such as mean age (51.80 ± 14.80 versus 49.9 ± 14.29 year) were comparable between mini-PCNL and RIRS.

## 4. The Results of Parameters in Meta-Analysis

### 4.1. Stone-Free Rate (SFR)

**Figure 2:**
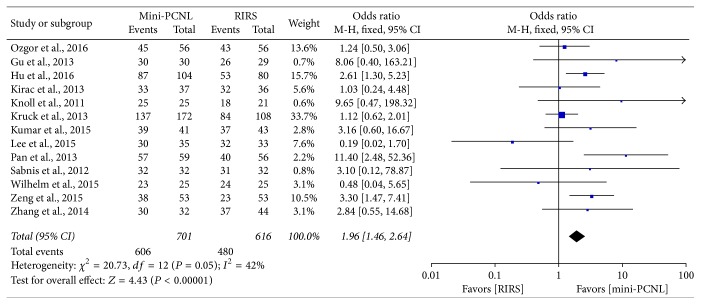
Forest plot and meta-analysis of stone-free rate in renal stone patients.

**Figure 3:**
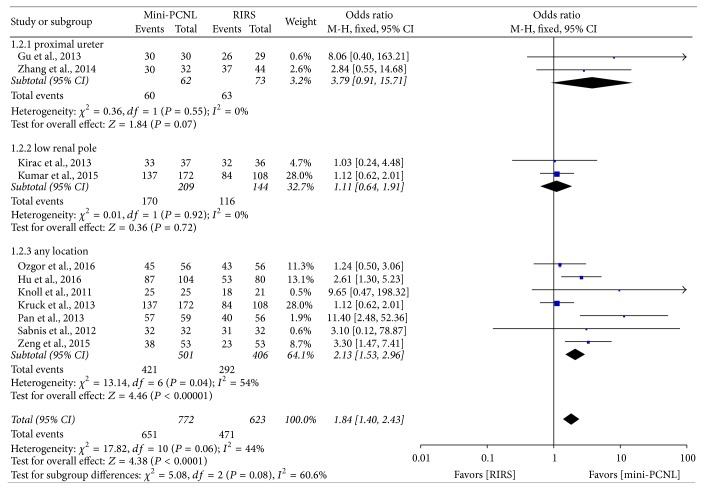
Forest plot and meta-analysis of location subgroup of stone-free rate.

**Figure 4:**
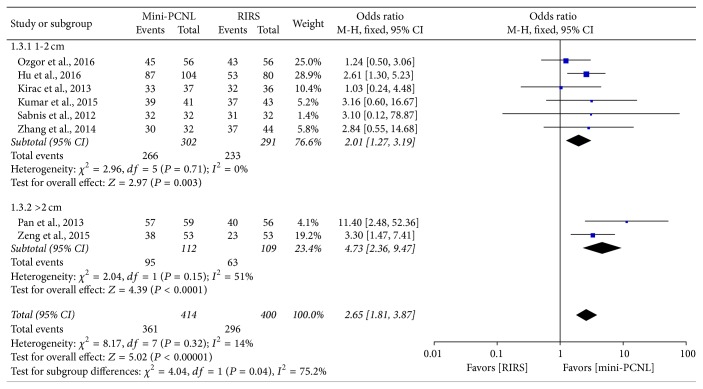
Forest plot and meta-analysis of stone size subgroup of stone-free rate.

SFR was evaluated with fixed-effect model, and the comparison of stone-free rate between the mini-PCNL and RIRS group is shown in Figure 2. All involved studies reported postoperative stone-free rate, and the result suggested that mini-PCNL group provided a significantly higher SFR than RIRS group (OR: 1.96; 95% CI, 1.46–2.64; *P* < 0.00001) with no significant homogeneity (*I*^2^ = 42%). As the stone location subgroup meta-analysis shows (Figure 3), mini-PCNL has significantly higher SFR than RIRS in any location (OR: 2.13; 95% CI, 1.53–2.96; *P* < 0.00001).  Figure 4 shows that mini-PCNL can lead to higher stone clearance in both 1-2 cm (OR: 2.01; 95% CI, 1.27–3.19; *P* = 0.003) and >2 cm subgroups (OR: 2.65; 95% CI, 1.81–3.87; *P* < 0.0001).

### 4.2. Operative Time

**Figure 5:**
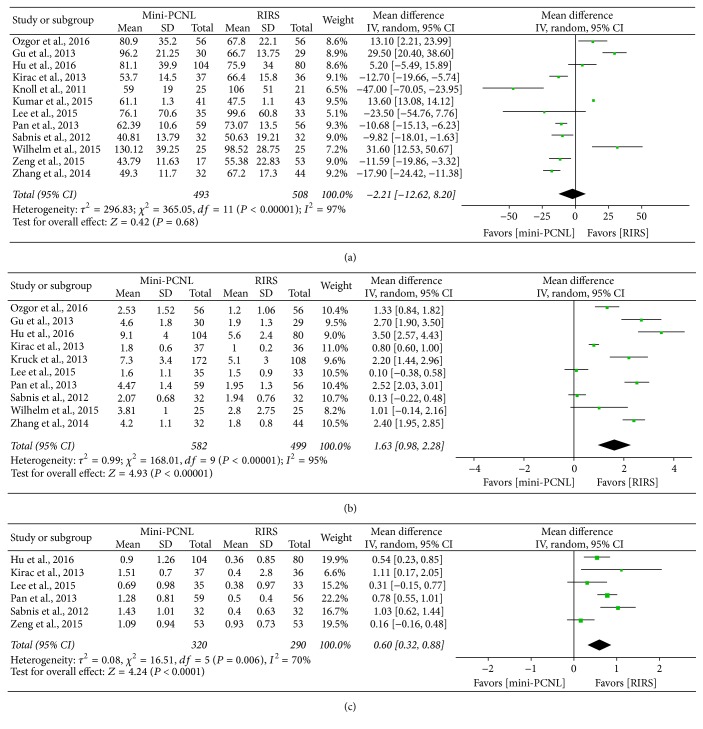
Forest plot and meta-analysis of outcomes in renal stone patients: (a) operative time; (b) hospital stay; (c) hemoglobin drop.

Twelve studies combined had reported operative time, which was evaluated with random effect model. As the meta-analysis result shows in Figure 5(a), there was no remarkable difference between mini-PCNL and RIRS (WMD, −2.21; 95% CI, −12.62–8.20; *P* = 0.68).

### 4.3. Hospital Stay

In 10 studies there were available data to extract in terms of hospital stay, which was analyzed by random effect model. As shown in Figure 5(b), hospital stay for RIRS is shorter than mini-PCNL (WMD: 1.63 d; 95% CI, 0.98–2.28; *P* < 0.00001).

### 4.4. Hemoglobin (Hb) Drop

Hb drop was analyzed by random effect model, and the result was shown in Figure 5(c). Six studies provided accessible data about Hb drop and the meta-analysis shows that RIRS led to less Hb drop than mini-PCNL (WMD, 0.60; 95% CI, 0.32–0.88; *P* < 0.0001).

### 4.5. Complication Rate

**Figure 6:**
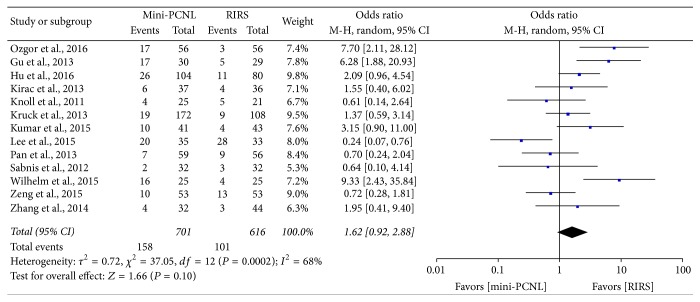
Forest plot and meta-analysis of total complications for two procedures.

**Figure 7:**
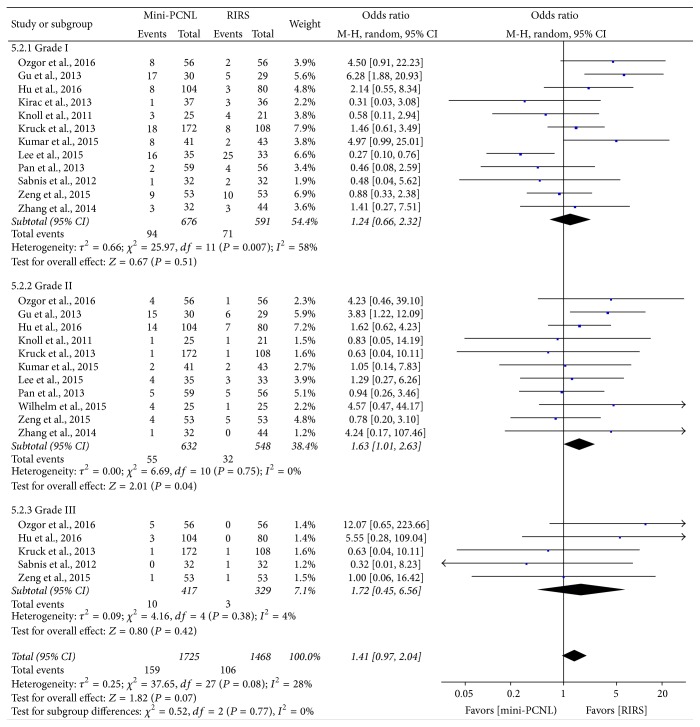
Forest plot and meta-analyses of postoperative complications.

All studies reported available data for the assessment of the complications between mini-PCNL and RIRS group. Random effect model and OR were used for statistical analysis and the result is shown in Figure 6. RIRS has a lower complication rate than mini-PCNL (OR: 1.62; 95% CI, 0.92–2.88; *P* = 0.10). Furthermore, we analyzed the complications according to Clavien-Dindo Classification (Table 4) to evaluate minor indisposition and major complication [28, 29]. As the results show, there is no significant difference between grade I and grade III complications between the two groups (OR: 1.24, 95% CI, 0.66–2.32, *P* = 0.51; OR: 1.41, 95% CI, 0.97–2.04, *P* = 0.77); however, we observed a significantly lower incidence of grade II complications in RIRS group (OR: 1.63; 95% CI, 1.01–2.63; *P* = 0.04) (Figure 7).

### 4.6. Sensitivity Analysis

The sensitivity analysis suggested that the results of this meta-analysis were relatively stable (Table 5). When only RCTs and high score non-RCTs (NOS: 7 of 9 points) were included, most of the outcomes including stone-free rate, operative time, total postoperative complications, and grade I and III surgery complications were not greatly changed. Meanwhile, significant differences of grade II complications, hemoglobin drop, and hospital stay between two groups were not found because of the reduced sample capacity. It is notable that even if the significant differences were no longer detectable in the sensitivity analysis, the tendency of meta-analysis stayed in the same direction.

### 4.7. Publish Bias Analysis

**Figure 8:**
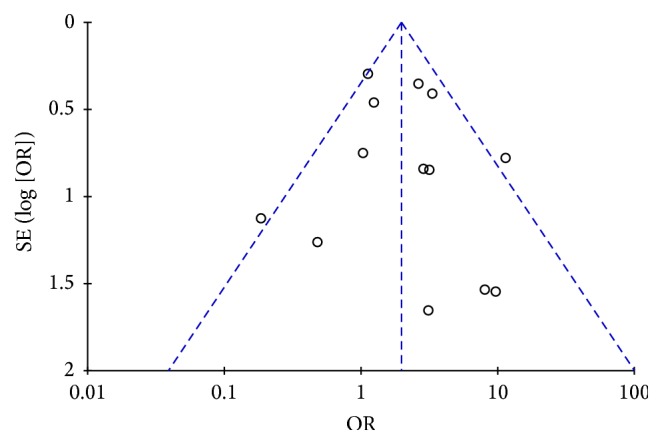
Funnel plot for the publication bias test of mini-PCNL versus RIRS.

The funnel plot (Figure 8) showed an apparent asymmetry, which suggested the existence of a potential publication bias.

## 5. Discussion

With high technological advancement, urologists who take charge of urolithiasis are in possession of high technique instruments, which leads to safer and more effective lithotripsy. So far PCNL is considered to be the recommended therapy for large stones > 2.0 cm by both AUA and EAU guidelines. Furthermore, with the development of the “mini-PCNL” procedure, smaller access sheaths (≤20 F) are becoming increasingly popular for its relative safety. Besides, recent reports suggested that RIRS is a safer approach that could lead to less complications and Hb drop than normal tract PCNL. We conducted this meta-analysis to systematically analyze the outcomes of two miniature procedures, mini-PCNL and RIRS, which cause considerably lesser trauma than standard PCNL, to find which one could lead to better efficacy and safety. And, to the best of our knowledge, this meta-analysis is an update analysis comparing these two modern minimally invasive approaches applying for upper urinary stone.

SFR is the most important parameter for estimating the efficacy of two approaches. According to the synthesis analysis of data, mini-PCNL has a higher SFR than RIRS group though there were various imaging modalities to identify. Stone-free rates are correlated with the lithotripsy and the location or size of stones. Seven inclusive studies used only laser to dispose stones and others made use of pneumatic or ultrasound waves to fragment calculi. Zhang et al. and Gu et al. included only proximal ureter stones and almost all included trails studied stones >10 mm. To evaluate the different locations that may impact SFR of two procedures, a subgroup analysis was performed. As Figure 3 shows, the proximal and low pole subgroups did not show any remarkable advantage of two approaches, while all locations showed that mini-PCNL has a significant advantage in SFR. Additionally, one stone size subgroup analysis was performed to estimate the impact on meta-analysis; results showed that mini-PCNL has more efficiency stone clearance in both 1-2 cm and >2 cm groups (Figure 4). Besides, mini-PCNL carries high efficiency quotient (EQ) (Table 6), which was related to SFR, percentage retreatment, and percentage requiring an auxiliary procedure (as the following formula) [30, 31], reported by three included studies (Table 5) [14, 16, 22]. However, De et al. had performed a meta-analysis that compared PCNL and RIRS for managing kidney stone and the results showed that RIRS can provide higher stone-free rates compared with mPCNL, which was opposite to our results [10]. It should be noted that only 5 literatures were included in the previous study, and the “mPCNL” in this study referred to micronephroscope which is 4.85 Fr and mini-PCNL from 11 to 19 Fr. This diversity of definition and sample size may result in the outcomes' difference between our meta-analysis and the previous study. Including more relative studies, the outcome would become more reliable.

(1) $$\begin{array}{c} {EQ}^{31}=\frac{precentage\;\;of\;\;stone\text{-}free}{100+precentage\;\;of\;\;retreatment+precentage\;\;of\;\;auxiliary\;\;procedure} \end{array}$$

Operative times were reported in 12 involved studies, and six studies indicated that mini-PCNL spent shorter operating time, while four studies favored RIRS. The overall meta-analysis showed that two procedures brought no significantly varied operation time; meanwhile, we noticed the heterogeneity in this section was as high as 97%, mainly led by Kumar et al., Gu et al., Ozgor et al., and Wilhelm et al. [12, 13, 16, 22]. If the four studies were excluded, heterogeneities would be declined to 70%, and the preference would favor mini-PCNL procedure. Operative time is closely related to nuance in surgical techniques and doctors' experience, different surgeons in different centers provided a large variation in operative time, and a significant heterogeneity was proved from twelve inclusive studies.

The overall analysis found that RIRS resulted in shorter hospital stay than mini-PCNL group. The reason for this difference might be less invasive caused by RIRS. Moreover, it carries lower complication rate and hemoglobin drop.

The size of the tract is one key factor for blood loss during endourology surgery, so mini-PCNL with miniature tract can reduce bleeding and the risk of blood transfusion compared to normal tract PCNL [32]. Besides, the overall analysis of the literature suggested that RIRS resulted in less hemoglobin drop than mini-PCNL. Accordingly, RIRS has a high efficiency for the management of intrarenal stones with a slight complication to patients [33, 34].

All trails have made the comparison of postoperative morbidity between mini-PCNL and RIRS. The results showed that RIRS provided a lower complication rate than mini-PCNL; however, the difference had no significance. The complications of mini-PCNL are similar to those of PCNL; bleeding, pain, and fever are very common [35–38].

Furthermore, we performed a subgroup meta-analysis of postoperative complications, classifying them into grades I, II, and III based on Clavien-Dindo Classification, between the two groups [28]. As Table 4 shows, grade I represents the morbidities that needed no pharmacological or surgical treatment, which could easily occur after operation, and grade III means complications requiring surgical, endoscopic, or radiological intervention, which rarely occur after lithotripsy operation. Thus, we did not observe a remarkable difference in comparison of grade I. Grade III complications were only observed in 5 studies, and the result showed that RIRS has a potential safety on severe complications. As for grade II complications, mini-PCNL has a significantly higher rate than RIRS according to our meta-analysis, which means RIRS was probably safer with respect to middle or severe morbidities after operation, and, in term of light deviations, the incidences of mini-PCNL and RIRS were similar.

There are several limitations in the present meta-analysis. In our systematic review and meta-analysis, we included the currently available comparative studies. Although we have done the sensitivity analysis to show that the results were relatively stable, there is still some bias of our conclusion caused by non-RCTs. Besides, heterogeneities among involved literatures, which may relate to diverse calculi size and location, different tract size, and lithotripsies, could lead to some limitations in our meta-analysis. In addition, most of the included trials failed to describe complications with the same criteria and blinding procedures in detail, and this might lead to conclusion bias, as the more details the literatures describe, the more credible the conclusion will be concluded. However, to the best of our knowledge, this study is one update review and meta-analysis to compare mini-PCNL and RIRS for treating renal calculi. We believe the results of the present meta-analysis could help urologists make better clinical decisions to manage stone disease patients.

## 6. Conclusions 

From this meta-analysis, we found that both mini-PCNL and RIRS can provide safe and effective treatment for renal calculi patients. In the light of these results, compared with RIRS, mini-PCNL provided significantly higher stone-free rate and efficiency quotient for management of upper urinary calculi, however, could increase the incidence of postoperative complications and the average hospital stay.

## Figures and Tables

**Table 1:** Summary of included studies.

Study	Institution (country)	Study period	Study design	LE	Inclusion criteria	Cases, *n*		Study quality
						mPCNL	RIRS	
Lee et al. [11]	Dongguk University Ilsan Hospital (South Korea)	2014-2015	RCT	2b	>1 cm, single or multiple renal stones	35	33	3^*∗*^
Ozgor et al. [12]	Haseki Teaching and Research Hospital (Turkey)	2012–2015	Retrospective case control	3b	1-2 cm, BMI > 30 kg/m^2^, any location	56	56	7^#^
Wilhelm et al. [13]	University Medical Centre Freiburg (Germany)	2013-2014	Matched-pair analysis	3b	1–3.5 cm	25	25	5^#^
Pan et al. [14]	Renji Hospital (China)	2005–2011	Prospective case control	3b	2-3 cm, single stone, any location	59	56	6^#^
Kirac et al. [15]	Koru Hospital (Turkey)	2009–2012	Retrospective case control	3b	<1.5 cm, lower pole	37	36	6^#^
Kumar et al. [16]	V.M. Medical College and Safdarjung Hospital (India)	2012-2013	RCT	2b	1-2 cm, single stone, lower pole	41	43	3^*∗*^
Sabnis et al. [17]	Muljibhai Patel Urological Hospital (India)	2009–2011	Prospective case control	3b	1-2 cm, single or multiple stones, any location	32	32	6^#^
Kruck et al. [18]	Multiple institutions (Germany)	2001–2007	Retrospective case control	3b	Any size, any location	172	108	4^#^
Hu et al. [19]	Tongji Hospital (China)	2010–2015	Retrospective case control	3b	1-2 cm, any location, older than 60 years old	104	80	6^#^
Zeng et al. [20]	The First Affiliated Hospital of Guangzhou Medical University (China)	2012–2014	Matched-pair analysis	3b	>2 cm, any location, solitary kidney	53	53	7^#^
Zhang et al. [21]	Wu Jieping Urology Center (China)	2010–2012	Prospective case control	3b	1-2 cm, single stone, proximal ureter	32	44	6^#^
Gu et al. [22]	Jiangsu Province Hospital (China)	2010-2011	RCT	2b	≥1.5 cm, proximal ureter	30	29	3^*∗*^
Knoll et al. [23]	Klinikum Sindelfingen-Boeblingen Medical Center (Germany)	2008–2010	Prospective case control	3b	1–3 cm, solitary renal calculi, any location	25	21	6^#^

LE = level of evidence; mPCNL = minimally invasive PCNL; RIRS = retrograde intrarenal surgery, RCT = randomized controlled trials.

^#^Using Newcastle–Ottawa Scale (score from 0 to 9).

^*∗*^Using Jadad scale (score from 0 to 5).

**Table 2:** Characters of patients and calculus.

Study	Treatment	Age (year)	Male/Female	BMI (kg/m^2^)	Stone size (mm)	Stone location (%)					
						Upper pole	Middle pole	Lower pole	Renal pelvis	Proximal ureter	Multiple
Lee et al. [11]	Mini-PCNL	59.3 ± 13.3	28/7	26.3 ± 3.9	39.1 ± 30.7	2.9	—	40.0	17.1	—	40.0
	RIRS	55.8 ± 11.2	28/5	25.6 ± 5.1	28.9 ± 17.5	3.0	—	30.3	27.3	—	39.4
Ozgor et al. [12]	Mini-PCNL	51.4 ± 14.3	25/31	34.0 ± 3.3	19.5 ± 3.9	8.9	1.8	26.8	25.0	—	37.5
	RIRS	54.2 ± 10.6	22/34	34.4 ± 5.0	18.3 ± 3.2	7.1	1.8	26.8	39.3	—	25.0
Wilhelm et al. [13]	Mini-PCNL	51.56 (15–75)	15/10	29.54 (18.75–42.94)	19.3 (10–35)	—	—	—	—	—	—
	RIRS	51.36 (19–77)	19/6	28.41 (18.4–38.57)	19.2 (10–35)	—	—	—	—	—	—
Pan et al. [14]	Mini-PCNL	49.37 ± 14.2	37/22	23.52 ± 3.7	22.37 ± 2.7	8.5	18.6	52.5	20.3	—	—
	RIRS	49.32 ± 13.7	36/20	23.69 ± 3.6	22.28 ± 2.6	12.5	12.5	51.8	23.2	—	—
Kirac et al. [15]	Mini-PCNL	41.02 ± 10.3	25/12	18.5 ± 4.9	10.5 ± 2.2	—	—	100	—	—	—
	RIRS	37.8 ± 8.7	22/14	18.3 ± 5.0	10.2 ± 2.9	—	—	100	—	—	—
Kumar et al. [16]	Mini-PCNL	33.7 ± 1.6	20/21	23.5 ± 1.2	13.3 ± 1.3	—	—	—	—	—	—
	RIRS	33.4 ± 1.4	20/23	23.6 ± 1.1	13.1 ± 1.1	—	—	—	—	—	—
Sabnis et al. [17]	Mini-PCNL	44.48 ± 12.36	19/13	—	15.2 ± 3.3	3.1	0.0	31.3	43.8	—	21.9
	RIRS	49.28 ± 12.19	25/7	—	14.2 ± 3.4	9.4	3.1	28.1	25.0	—	34.4
Kruck et al. [18]	Mini-PCNL	53.3 ± 14.8	109/63	—	12.6 ± 9.5	—	—	42.7	—	—	—
	RIRS	50 ± 16.7	69/39	—	6.8 ± 6.9	—	—	76.8	—	—	—
Hu et al. [19]	Mini-PCNL	65.5 ± 4.9	56/48	23.7 ± 3.5	15.8 ± 3.4	1.9	7.7	13.5	40.4	36.5	59.6
	RIRS	65.1 ± 5.2	45/35	23.0 ± 3.1	15.8 ± 3.4	3.8	12.5	17.5	37.5	28.8	57.5
Zeng et al. [20]	Mini-PCNL	53.04 ± 14.09	36/17	23.26 ± 3.41	329.34 ± 184.27^*∗*^	3.8	3.8	22.6	22.6	—	47.2
	RIRS	48.47 ± 11.96	39/14	23.63 ± 3.83	331.87 ± 182.55^*∗*^	5.7	3.8	18.9	26.4	—	45.3
Zhang et al. [21]	Mini-PCNL	42.7 ± 13.6	24/8	—	15.6 ± 2.5	—	—	—	—	100	—
	RIRS	43.3 ± 11	29/15	—	14.9 ± 2.3	—	—	—	—	100	—
Gu et al. [22]	Mini-PCNL	42.5 ± 10.1	—	—	17.27 (15–25)	—	—	—	—	100	—
	RIRS	44.22 ± 13	—	—	16.23 (15–25)	—	—	—	—	100	—
Knoll et al. [23]	Mini-PCNL	56 ± 13	15/10	27 ± 5	18 ± 5	4.0	68.0	12.0	56.0	—	—
	RIRS	53 ± 11	9/12	31 ± 7	19 ± 4	9.5	66.7	4.8	38.1	—	—

*Note.* Mini-PCNL = minimally invasive percutaneous nephrolithotomy; RIRS = retrograde intrarenal surgery; Unit = mm^2^; all other units are in millimeters.

**Table 3:** The characters of the surgical methods of included studies.

Study	Treatment	Access sheath size, Fr	Dilator	Nephroscope size	Lithotripsy
Lee et al. [11]	Mini-PCNL	18	Balloon	15 F	Laser
	RIRS	14/16	UAS	7.5 F	Laser
Ozgor et al. [12]	Mini-PCNL	18 or 20	Amplatz	17 F	Laser and ultrasound
	RIRS	19/23	UAS	7.5 F	Laser
Wilhelm et al. [13]	Mini-PCNL	10 and 14	PTFE dilators/Amplatz	13 F	Laser
	RIRS	7/8	UAS	—	Laser
Pan et al. [14]	Mini-PCNL	18	Amplatz	14 F	Laser
	RIRS	12	UAS	Olympus P3 or P5	Laser
Kirac et al. [15]	Mini-PCNL	20	Amplatz	15–16.5 F	Pneumatic or ultrasound energy
	RIRS	9.5/11.5 or 12/14	UAS	8 or 9.5 F,	Laser
Kumar et al. [16]	Mini-PCNL	18	gauge needle	15 F	Pneumatic
	RIRS	12	UAS	8/9.8 F	Laser
Sabnis et al. [17]	Mini-PCNL	16–19	22-gauge Skinny Needle	15/18 F and 16.5/19.5 F	Laser
	RIRS	14	UAS	7.5-F Flex X–2	Laser
Kruck et al. [18]	Mini-PCNL	16–18	Metal	12 F	Ultrasound
	RIRS	—	Fascial dilator	Flex-X/Flex-X2	Laser
Hu et al. [19]	mPCNL	16–20	Fascial dilator	8/9.8 F	Laser
	RIRS	12/14	UAS	Flex-X2	Laser
Zeng et al. [20]	Mini-PCNL	18	Fascial dilators	—	Laser and pneumatic
	RIRS	12/14	UAS	7.5 F	Laser
Zhang et al. [21]	Mini-PCNL	18–20	facial dilators	8.6/9.8 F	Laser and pneumatic
	RIRS	12/14	UAS	5.3–8.4 F	Laser
Gu et al. [22]	Mini-PCNL	12/18	Fascial dilators	8.5/9.8 F	Laser
	RIRS	—	UAS	7.4 F	Laser
Knoll et al. [23]	Mini-PCNL	18	Amplatz	14 F	Laser
	RIRS	12/14	—	—	Laser

Mini-PCNL = minimally invasive percutaneous nephrolithotomy; RIRS = retrograde intrarenal surgery; UAS = ureteral access sheath placement.

**Table 4:** Clavien-Dindo Classification for surgical complication.

Surgical complications classification	Description	For example
Grade I	Any deviation from the normal postoperative course without the need for pharmacological treatment or surgical, endoscopic, and radiological interventions.	Bleeding, pain, fever, vomiting, tachycardia, hyperkalemia, and so forth.
Grade II	Requiring pharmacological treatment with drugs other than such allowed for grade I complications.	Minor pelvic/ureter perforation, hypertension requiring nicardipine, urinary tract infection, subcapsular hematoma, and so forth.
Grade III	Requiring surgical, endoscopic, or radiological intervention.	Embolization, steinstrasse, and so forth.
Grade IV	Life-threatening complication (including CNS complications)^‡^ requiring IC/ICU-management	Shock and so forth.
Grade V	Death of a patient.	Death and so forth.

**Table 5:** Sensitivity analysis results.

Items	Studies	Sample size	Tests for heterogeneity		Analysis model	Test for overall effect	RR/WMD 95% CI		Favors
		Mini-PCNL/RIRS	*I* ^2^	*P* value		*Z*	*P* value		
Stone-free rate	[11, 12, 16, 20, 22]	215/214	51%	0.09	Fixed	2.52	0.01	1.92 [1.16, 3.18]	Mini-PCNL
Operative time	[11, 12, 16, 20, 22]	215/214	94%	<0.00001	Random	1.06	0.29	7.60 [−5.32, 20.53]	RIRS
Hemoglobin drop	[11, 20]	88/86	0	0.60	Random	1.55	0.12	0.21 [−0.06, 0.47]	RIRS
Hospital stay	[11, 12, 22]	121/118	94%	<0.00001	Random	1.99	0.05	1.34 [0.02, 2.67]	RIRS
Total complication	[11, 12, 16, 20]	185/185	84%	0.00003	Random	0.44	0.66	1.38 [0.33, 5.84]	RIRS
Grade I	[11, 12, 16, 20]	185/185	78%	0.04	Random	0.44	0.66	1.35 [0.35, 5.15]	RIRS
Grade II	[11, 12, 16, 20]	185/185	0	0.65	Random	0.46	0.64	1.22 [0.52, 2.86]	RIRS
Grade III	[12, 20]	109/109	35%	0.21	Random	0.94	0.35	3.36 [0.27, 41.48]	RIRS

Mini-PCNL: minimally invasive percutaneous nephrolithotomy; RIRS: retrograde intrarenal surgery; RR: relative risk; WMD: weighted mean difference; CI: confidence interval.

**Table 6:** Efficiency quotient in included studies.

Study	EQ for mini-PCNL	EQ for RIRS	*P* value
Pan et al. [14]	0.904	0.523	—
Kumar et al. [16]	0.915	0.842	0.01
Gu et al. [22]	0.830	0.500	—

EQ = efficiency quotient.
